# Critical threshold target attainment rates for tazobactam combined with piperacillin among patients admitted to the ICU with hospital-acquired pneumonia

**DOI:** 10.1128/aac.01766-25

**Published:** 2025-12-29

**Authors:** Ryan Williamson, Marta Zurawska, Adrian Valadez, Emma Harlan, Marc H. Scheetz, Michael N. Neely, Paul R. Yarnold, Mengjia Kang, Helen K. Donnelly, Franciso Martinez, Erin Korth, Rachel L. Medernach, Sophia H. Nozick, Alan R. Hauser, Egon A. Ozer, Estefani Diaz, Alexander V. Misharin, Richard G. Wunderink, Nathaniel J. Rhodes

**Affiliations:** 1 Department of Pharmacy Practice, Midwestern University, College of Pharmacy 15475, Downers Grove, Illinois, USA; 2 Pharmacometrics Center of Excellence, Midwestern University 69281, Downers Grove, Illinois, USA; 3 Department of Pharmacy, Northwestern Memorial Hospital 24560, Chicago, Illinois, USA; 4 Departments of Pharmacology and Biomedical Sciences, College of Graduate Studies, Midwestern University 69281, Downers Grove, Illinois, USA; 5 Keck School of Medicine, University of Southern California 5116, Los Angeles, California, USA; 6 Laboratory of Applied Pharmacokinetics and Bioinformatics, The Saban Research Institute, Children's Hospital of Los Angeles 466592, Los Angeles, California, USA; 7 Optimal Data Analysis, LLC, Chicago, Illinois, USA; 8 Division of Pulmonary and Critical Care Medicine, Department of Medicine, Northwestern University Feinberg School of Medicine 166943, Chicago, Illinois, USA; 9 Division of Infectious Diseases, Department of Internal Medicine, RUSH University Medical Center 2468, Chicago, Illinois, USA; 10 Department of Microbiology-Immunology, Northwestern University Feinberg School of Medicine 547641, Chicago, Illinois, USA; 11 Division of Infectious Diseases, Department of Medicine, Northwestern University Feinberg School of Medicine 166943, Chicago, Illinois, USA; 12 Center for Pathogen Genomics and Microbial Evolution, Havey Institute for Global Health, Feinberg School of Medicine, Northwestern University 12244, Chicago, Illinois, USA; 13 Robert H. Lurie Comprehensive Cancer Research Center, Feinberg School of Medicine, Northwestern University 12244, Chicago, Illinois, USA; Providence Portland Medical Center, Portland, Oregon, USA

**Keywords:** population pharmacokinetics, tazobactam, target attainment, renal dysfunction

## Abstract

Optimizing antibiotic exposures in hospital-acquired pneumonia (HAP) is crucial; however, dosing decisions often overlook target levels for the beta-lactamase inhibitor tazobactam (TAZ), and the impact of renal dysfunction and renal replacement therapy on TAZ pharmacokinetics (PK) remains poorly described. We developed a population PK model of TAZ using 162 plasma samples from 35 ICU patients with HAP, including those receiving continuous renal replacement therapy (CRRT). TAZ concentrations were quantified using validated LC-MS/MS methods. A one-compartment model was fit using Monolix, with clearance modeled as a function of creatinine clearance, CRRT effluent flow rate, and intermittent hemodialysis. Monte Carlo simulations assessed the probability of unbound TAZ concentrations exceeding 1, 2, or 4 mg/L for 100% of the interval between 24 and 48 h across intermittent, extended, and continuous infusion (CI) regimens, stratified by renal function and CRRT. TAZ clearance was driven by renal function and CRRT flow rate. All regimens achieved ≥90% probability of target attainment (PTA) at thresholds of 1–2 mg/L. At 4 mg/L, PTA fell below 90% in patients with creatinine clearance ≥150 mL/min for all regimens, with low-dose CI performing the worst. High-dose CI improved PTA at this higher threshold but may increase the risk of piperacillin overexposure. TAZ exposures sufficient for enzymatic beta-lactamase inhibition are generally achieved with standard dosing for lower thresholds but not for more aggressive targets (e.g., high extended-spectrum β-lactamase expression), particularly in patients with preserved or augmented renal function, who may require therapeutic drug monitoring or alternative therapy.

## INTRODUCTION

Patients experiencing ventilator-associated pneumonia (VAP) are at increased risk of death (1, 2). Contemporary clinical trials evaluating broad-spectrum β-lactams for the treatment of hospital-acquired pneumonia (HAP) and VAP reported that infections due to Gram-negative Enterobacterales account for over half of pathogens identified in this setting (3–5). β-Lactamases, including extended-spectrum β-lactamases (ESBLs), that are carried by Enterobacterales spp. can reduce the effectiveness of first-line antibiotic treatments, such as piperacillin-tazobactam (TZP) compared with meropenem (6–8). Among Enterobacterales isolates collected from nosocomial pneumonia patients at baseline in recent trials, between 30% and 45% were ESBL-producing (3–5). The β-lactamase inhibitor tazobactam (TAZ) has previously demonstrated concentration and time-dependent inhibition of beta-lactamase enzymes both *in vitro* and *in vivo*. An *in vitro* study by Nicasio et al. found that TAZ demonstrated a *f*T_>threshold_ inhibitory effect on an ESBL (CTX-M) beta-lactamase, which enhanced killing in combination with piperacilin (PIP) (9). Among isogenic *E. coli* strains expressing different levels of CTX-M, a 2-log kill was achieved vs the high-expressing isolate when unbound (free) TAZ concentrations exceeded a threshold of 2 mg/L for at least 85% of the dose interval (9). Likewise, in a hollow-fiber experiment, TAZ restored bacterial killing in combination with PIP vs an isogenic *E. coli* strain expressing TEM-3 β-lactamase when %T_>4 mg/L_ was 38%–50% (10). TAZ has also demonstrated *f*T_>threshold_ exposure-response effects in animal models evaluating *E. coli* expressing TEM-1 β-lactamase. Rodriguez et al. found that achieving 42%–56% *f*T_>threshold_ for TAZ at a threshold of 2 mg/L was associated with a 1 log-kill in combination with PIP in a murine thigh infection model (11). Thus, optimizing TAZ exposures in combination with PIP may improve bacterial killing during empiric treatment of patients with HAP and VAP caused by Enterobacterales.

Whereas traditional package insert dosing for TZP for nosocomial pneumonia is based on intermittent infusion (II) dosing over 30 min in combination with an aminoglycoside (12), contemporary TZP dose regimens are often given as monotherapy and administered as an extended infusion regimen (EI) dosed over 3 or 4 h. The use of continuous infusion (CI) as an optimization approach has also gained greater attention in the light of the BLING III trial (13). Prolonged and continuous dosing of β-lactam antibiotics has been associated with improved rates of clinical cure and lower odds of mortality vs II dosing in patients with sepsis, septic shock, and greater severity of illness (13–16). Prior research focused on optimization of PIP pharmacokinetics (PK)/pharmacodynamics (PD) in the setting of EI and CI, with few studies evaluating joint target attainment for both β-lactam and the β-lactamase inhibitor. Cojutti et al. evaluated the ratio of PIP to TAZ in real-world patients with various degrees of renal function and found that median ratios varied from 4.8 to 11.3 across renal dispositions (17). They defined joint optimal exposure as having a free steady-state concentration relative to the breakpoint MIC ratio for PIP ≥4 and at the same time having a free steady-state concentration relative to a critical threshold concentration ratio for TAZ ≥1, although outcomes were not available in their study (17). Gatti et al. applied the same definition of optimal joint exposure for PIP and TAZ in a cohort of 43 pneumonia patients receiving CI TZP as monotherapy: they found that jointly optimal PIP and TAZ target attainment reduced microbiological failure (OR, 0.03; 95% CI, 0.003–0.27), whereas sub-optimal exposure increased microbiological failure in their multivariable model (18). Given the variability in site of infection PK/PD attainment in critically ill patients, and signals for improved microbiological outcomes when TAZ remains above a threshold concentration (18), evaluation of 100% *f*T_>threshold_ for thresholds up to 4 mg/L may help guide clinical dose optimization efforts.

We recently developed a population PK model of PIP (19) and TAZ (reported herein) among critically ill patients with HAP. In the present study, we apply our TAZ model to evaluate target attainment for II, EI, and CI dosing strategies to evaluate the probability of adequate TAZ exposures in pneumonia patients across a range of doses and renal dispositions.

## MATERIALS AND METHODS

### Patients

Critically ill patients with HAP admitted to the Medical ICU at Northwestern Memorial Hospital were enrolled between June 2018 and May 2023 and received PTZ dosed according to institutional protocols based on renal function and indication (asp.nm.org).

In this study, residual blood samples left over from clinical care were opportunistically captured from patients enrolled in the Successful Clinical Response in Pneumonia Therapy (SCRIPT, U19AI135964) (https://script.northwestern.edu/database/) and RX-SCRIPT studies, which focused on β-lactam-treated patients with and without continuous renal replacement therapy (CRRT) (R21AI174159 and R01AI158530, respectively). Collection times were recorded from the electronic medical record for residual blood samples. Patients requiring concurrent extracorporeal membrane oxygenation (ECMO) were excluded. All patients or their legally authorized representative provided written informed consent prior to being enrolled in SCRIPT and RX-SCRIPT. The study protocol was approved by the Northwestern University Institutional Review Board (STU002048680). Plasma samples were processed according to protocol and stored for batch PK analysis at −80°C (20).

### Bioanalysis of drug concentrations

Total TAZ was quantified using Agilent 1260 Infinity II liquid chromatography system coupled with Agilent Ultivo Triple Quadrupole mass spectrometer (LC-MS). The analytical separation was achieved using an Agilent Infinity Lab Poroshell 120 EC-C18 column (100 mm × 3.0 mm × 2.7 μm). Mobile phase A was 0.1% formic acid in water, and mobile phase B was 100% LC/MS-grade acetonitrile. Transitions (*m*/*z*) for TAZ were quantitation 299 → 137.9, qualification 299 → 67.9; TAZ-^15^N_3_ (*m*/*z*: 302 → 137.9 and 70.8) served as an internal standard. The assay was linear from 0.5 to 80 mg/L; ALQ samples were diluted to fall within the linear range. Accuracy (inter-day: 97.1%–104%; intra-day: 89.3%–119%) and precision (inter-day coefficient of variation [CV%]: 2.17%–15.2%; intra-day CV%: 1.01%–6.4%] met Food and Drug Administration (FDA) requirements for bioanalytical method validation (21). TAZ concentrations below the lower limit of quantitation (LLOQ) of 0.5 mg/L were considered below the limit of quantitation (BLQ) and modeled in Monolix using interval censoring, with the likelihood estimated over the interval [0, LLOQ]. PIP in plasma was analyzed as described by Zurawska et al. (19).

### Population PK modeling

Population PK) modeling was performed using Monolix 2024R1. The system was described using a series of differential equations. Base one-compartment and two-compartment models were evaluated, with first-order elimination from the central compartment. Given the opportunistic nature of blood sampling (typically collected in the morning) relative to hemodialysis (HD) times (typically conducted in the late afternoon), TAZ HD clearance was fixed to literature values (22). Improvements in model fitness were evaluated in Sycomore 2024R1. The piperacillin population PK model is described by Zurawska et al. (19).

### Covariate, regressor, and error models

Due to the opportunistic nature of PK sampling over a long time horizon, which did not strictly align to specific occasions across subjects, we chose to use the regressor approach available within Monolix to model time-varying covariates. Planned regressors evaluated included total body weight on Vd, CrCL on CL, and effluent flow rate on clearance in CRRT. Planned covariates evaluated included age, body surface area (BSA), and sex. Multiple clearance pathways, non-CRRT, CRRT, and HD clearances were evaluated *a priori*. In the base model, total clearance was described by the following piece-wise equations:


(1) $$CL_{total}=CL_{nonCRRT}+CL_{CRRT}+CL_{HD}$$

(2) $$CL_{nonCRRT}=CL_{renal}+CL_{nonrenal}$$

where CL_renal_ was scaled to CrCL, and CL_nonrenal_ was a random intercept.

Covariate models considered continuous variables (e.g., age, weight, body surface area) using power models, for example:


(3) $$CL_{individual\left(i\right)}=CL_{population}\times {\left(\frac{Covariate_{i}}{Referent}\right)}^{beta\_covariate}\times e^{\eta CL,i}$$

where ηCL,i was the random effect estimate for clearance.

Fractional changes from the referent category were evaluated using an indicator variable for categorical variables (e.g., sex), for example:


(4) $$V_{individual\left(i\right)}=\;V_{population}\times \left(1-Covariate_{i}\times beta\_covariate\right)\times e^{\eta V,i}$$

where ηV,i was the random effect estimate for volume.

Covariates and regressors were evaluated sequentially with a change in the objective function value of 3.84 required for inclusion and 6.64 for backward elimination. Regressors were considered time-varying covariates (e.g., CrCL, CRRT, HD) and were integrated into the base model. Final model selection was determined on the basis of minimization of the corrected Bayesian Information Criterion, model diagnostics, including condition number and the rule of parsimony. Time after dose visual predictive checks (VPCs) (23) were generated in R using observed and simulated (*n* = 500 replicates) TAZ concentration-time profiles (six equal sized bins were used). Observed concentrations that were BLQ (i.e., censored) were excluded from percentile calculations. Uncertainty in the final model parameters was quantified using non-parametric (*n* = 1,000) bootstrap resamples.

### Simulations

The final covariate-adjusted population model was translated into Simulx 2024R1. Monte Carlo simulations were conducted to assess the PK/PD target attainment 48 h into a renal-adjusted CI dosing regimen after administration of a 500 mg loading dose (LD). Renal function was categorized based on the Cockcroft-Gault equation (24), with dose adjustments made at Cockcroft-Gault (24) calculated CrCL cutoff values of 25, 50, 75, and 150 mL/min. For patients receiving CRRT, effluent rates of 25 and 35 L/kg/h were simulated, assuming a measured total body weight of 70 kg. We also evaluated effluent flow rates based on larger body weights (e.g., 91 and 126 kg) in a sensitivity analysis. Simulations evaluated free (i.e., unbound) TAZ concentrations using published protein binding correction of 70% (12). The probability of free TAZ plasma concentrations exceeding 1, 2, and 4 mg/L threshold values for 100% of the interval between 24 and 48 h after the start of treatment was evaluated as a conservative PK/PD target for patients with pneumonia (25). For each dose regimen and renal disposition (as shown in Table S2), 1,000 simulated patients were modeled using the final model population parameters and their uncertainties. CI regimens were evaluated as low dose (375–1,125 mg/day IV over 24 h) or high dose (750–1,500 mg/day) based on renal disposition. EI regimens (375–500 mg IV every 8–12 h over 4 h) and II regimens (375–500 mg IV every 6 h over 30 min) were designed to approximate HAP dosing across renal states. Simulation results were visualized using the *ggplot2* package for R (version 4.4.2).

## RESULTS

A total of 162 plasma samples were available from 35 patients with pneumonia (mean age 62 years, 51% female, mean body weight 79.6 kg). Complete demographic data for these patients are described in the manuscript by Zurawska et al. (19). Of the 162 samples, four were BLQ. A total of 16 patients required RRT during PIP treatment, of which 15 required CRRT and one required HD. Among patients requiring CRRT, the highest mean ± SD effluent flow rate was 2.75 ± 1.1 L/h (32 ± 7.8 mL/kg/h). Among patients not requiring RRT, the mean ± SD creatinine clearance was 78 ± 68 mL/min (range: 9 to 229 mL/min).

### Base model development and model selection

Base model development and covariate evaluations are described in depth by Zurawska et al. (19) and summarized in Table S1. Ultimately, a one-compartment PK model structure was selected with total TAZ CL equal to the sum of renal CL and non-renal clearance among patients not receiving CRRT and equal to CRRT or HD CL when on renal replacement. PIP and TAZ clearance were modeled independently. Attempts to model TAZ disposition and clearance as a scalar of PIP parameters resulted in imprecision in inter-individual variability estimates (data not shown).

### Covariate model

Clearance of TAZ was modeled as a function of renal function (measured via creatinine clearance, CrCL), dialysis status (intermittent hemodialysis, HD), and CRRT with varying effluent flow rates. The final covariate-adjusted population PK model equations were:


(5) $$CL_{nonCRRT_{ind}}=\left(CL_{CrCL_{pop}}\times \left(\frac{CrCL}{120mL/min}\right)+CL_{NR_{pop}}\right)\times \left(1-CRRT\right)\times \left(1-HD\right)$$

(6) $$CL_{CRRT_{ind}}=CL_{CRRT_{pop}}\times \left(\frac{FLOW}{2L/hr}\right)\times CRRT$$

(7) $$CL_{HD_{ind}}=CL_{HD_{pop}}\times HD$$

In these equations, HD and CRRT are indicator variables (either on or off), and FLOW was the CRRT effluent flow rate in mL/h. Intra-HD clearance for TAZ was taken from the literature and fixed at 5.68 L/h (22).

### Final population PK model parameters

**Fig 1:**
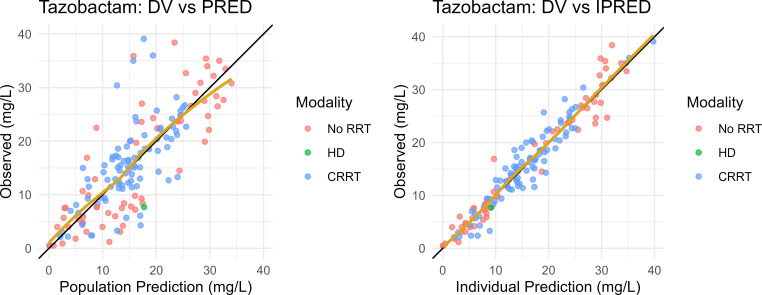
Goodness-of-fit plots for the final population PK model of TAZ. Observed plasma concentrations of TAZ (DV) on the y-axis to model-predicted concentrations on the x-axis. Left: Population PRED. Right: Empirical Bayes Estimates (iPRED). Goldenrod line: loess spline. Patients are stratified by renal replacement status and modality.

**Fig 2:**
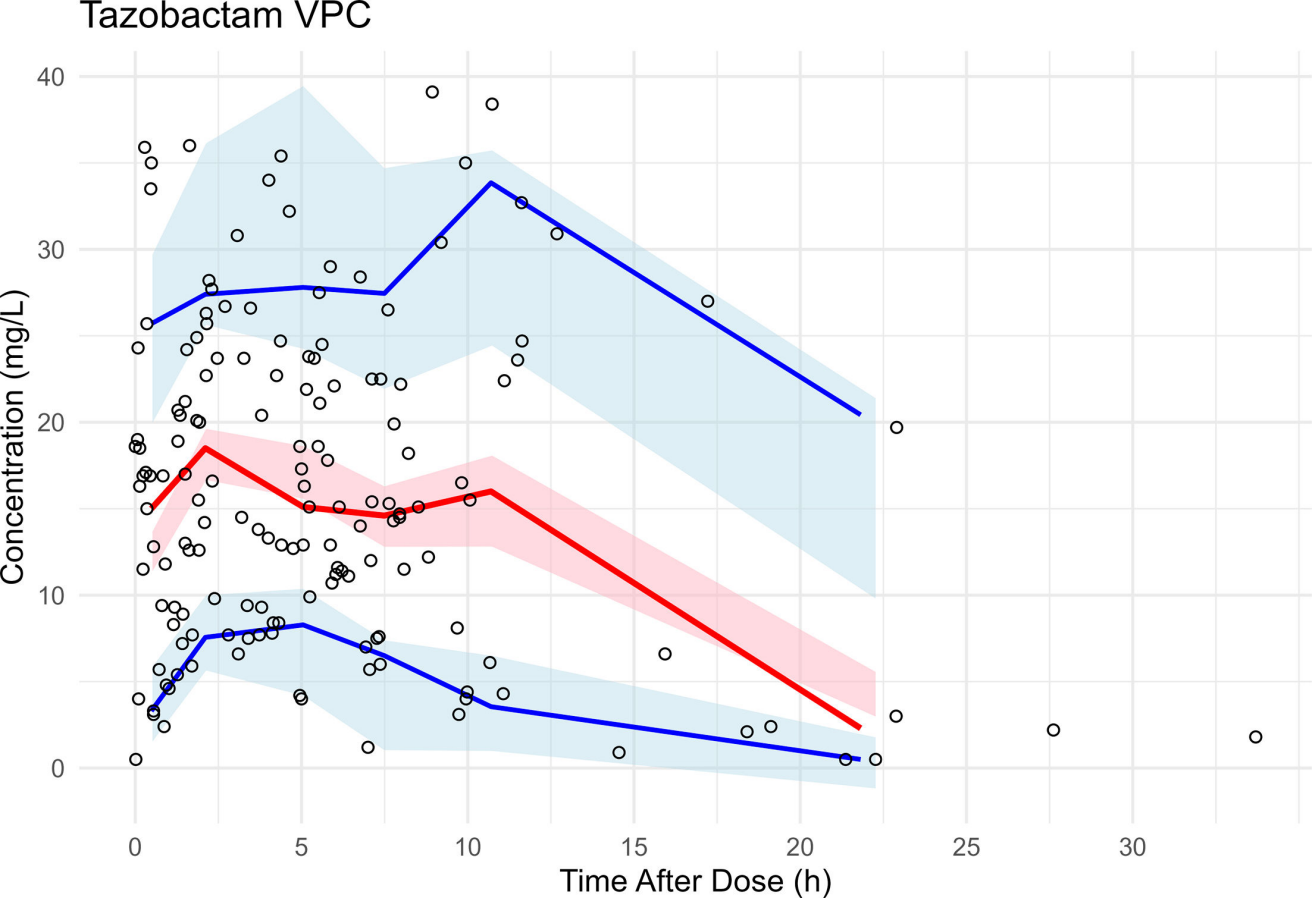
Visual predictive check for the final TAZ population PK model. Prediction-corrected visual predictive check (VPC). Shaded ribbons show the prediction-corrected simulated concentrations from the final model across 500 replicates: blue = 10th–90th percentile, pink = interquartile range. Solid lines show the median (red) and 10th/90th percentiles (blue) of the observed, prediction-corrected concentrations. Open circles: observations.

The final PK model parameter estimates are shown in Table 1. The population typical estimates for TAZ Vd, CL_R_, and CL_NR_ were 32.8 L, 4.9 L/h, and 0.91 L/h, respectively. For patients on CRRT, the population typical estimates for TAZ CL were 4 L/h based on a 2 L/h effluent flow rate. Inter-individual variability (IIV) estimates were reasonable (RSE: 24%–58%) with CV% for Vd, CL_R_, and CL_CRRT_ of 19%, 127%, and 26%, respectively (Table 1). Shrinkage ranged from low (23% CL_renal_) to moderate (46% CL_CRRT_) for clearance estimates and was elevated for Vd (54%). Goodness-of-fit plots for the population and Bayesian posterior predictions TAZ model are shown in Fig. 1. The model yielded acceptable predictive performance in pc-VPC analysis (Fig. 2).

**TABLE 1:** Population model parameters and estimates for TAZ^*a*^^,^^*b*^

Parameter	Estimate, mean	Non-parametric bootstrap resampling (*n* = 1,000), median	RSE (%)	Non-parametric bootstrap resampling (*n* = 1,000)	
				Lower 2.5th CI	Upper 97.5th CI
**Fixed effects**					
Vd, L	32.8	32.7	7.5%	28.6	38.1
CL_CrCL_, L/h/120 mL/min	4.9	5.3	27.4%	3.0	8.0
Non-renal CL, L/h	0.91	0.83	22.1%	0.33	1.35
CL_HD_, L/h	5.68	Fixed	Fixed	Fixed	Fixed
CL_CRRT_, L/h	4.0	4.0	8.0%	2.9	4.1
**Interindividual variability**					
**Random effects**	**C.V. %**				
ω_Vd_	19.0%	16.1%	45.2%	5%	35%
ω_CLCrCL_	127%	96.7%	23.6%	52.1%	200%
ω_CLCRRT_	26.1%	23.5%	57.9%	2.7%	46.2%
Residual error					
Additive (mg/L)	1.6	1.46	53.2%	3.6E-4	2.91
Proportional error (%)	6.4%	7.2%	67.0%	0.02%	19.2%

^*a*^ Population mean PK parameters and associated uncertainties for TAZ. Fixed effects reported as mean estimate; interindividual variation (ω) reported as CV % based on the standard deviation of the random effects. TAZ clearance was estimated as a piecewise time-varying clearance model with hemodialysis CL fixed, and renal (CrCL), non-renal, and CRRT CL estimated. Upper 97.5th and lower 2.5th percentiles from bootstrap replicates.

^*b*^ CL, clearance; CRRT, continuous renal replacement; CrCL, Cockcroft Gault estimated creatinine clearance; HD, hemodialysis; Vd: volume of distribution.

### Simulations

**Fig 3:**
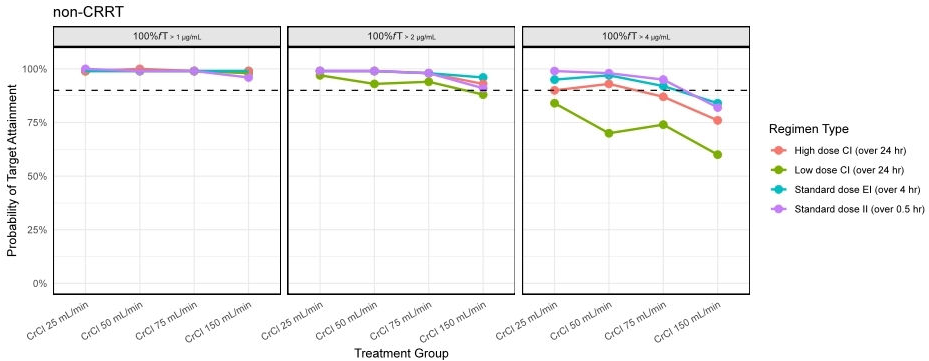
Probability of simulated unbound TAZ concentrations exceeding threshold values for II, EI, and CI by CrCL. Probability of simulated unbound TAZ concentrations achieving 100% *f*T_ > threshold_ for threshold targets of 1, 2, and 4 mg/L for II, EI, and CI regimens across renal function strata from 24 to 48 h after treatment is started. A dashed horizontal line indicates 90% target attainment. CI regimens are grouped as low (375–1,125 mg/day) or high (500–1,500 mg/day) dose. EI regimens (375–500 mg IV every 8–12 h over 4 h) and II regimens (375–500 mg IV every 6 h over 0.5 h) were designed to approximate HAP dosing across renal states.

Simulation revealed that, after a LD and 24 h of treatment, all dosing regimens achieved ≥90% target attainment for 100% *f*T_>threshold_ for a threshold of 1 mg/L (Fig. 3) on day 2 of treatment in patients not on CRRT. Similarly, all regimens achieved ≥90% target attainment for 100% *f*T_>threshold_ for a threshold of 2 mg/L except the TAZ regimen of 1,125 mg/day given as a CI in patients with a CrCL of 150 mL/min, where the probability of attainment fell to 88%. In contrast, at a target of 100% *f*T_>threshold_ of 4 mg/L all simulated CI regimens fell below 90% attainment at a CrCL ≥ 75 mL/min, and all regimens failed to achieve the target for patients with CrCL = 150 mL/min. The low-dose CI regimens failed to achieve ≥90% target attainment across all renal dispositions at a threshold target of 4 mg/L, whereas the high-dose CI regimens generally performed better (Fig. 3). Based on low- and high-dose CI simulations of PIP conducted by Zurawska et al. (19), joint attainment of PIP concentrations between 16 and 96 mg/L and 100% *f*T_>threshold_ for TAZ at a threshold of 2 mg/L was best achieved using the low dose CI regimen in patients up to a CrCL of 75 mL/min. However, when optimizing for 100% *f*T_>threshold_ at a threshold of 4 mg/L, a high-dose CI regimen would be preferred to a low-dose regimen up to a CrCL of 50 mL/min, at the cost of an increased probability of exceeding an upper bound PIP target of 96 mg/L. Among patients not on CRRT, II and EI dose regimens provided ≥90% attainment for a TAZ target of 100% *f*T_>threshold_ for a threshold of 4 mg/L up to a CrCL of 150 mL/min, suggesting a potential role for EI-based dose regimens in combination with therapeutic drug monitoring for patients with preserved (i.e., CrCL ≥ 75 mL/min) renal function.

**Fig 4:**
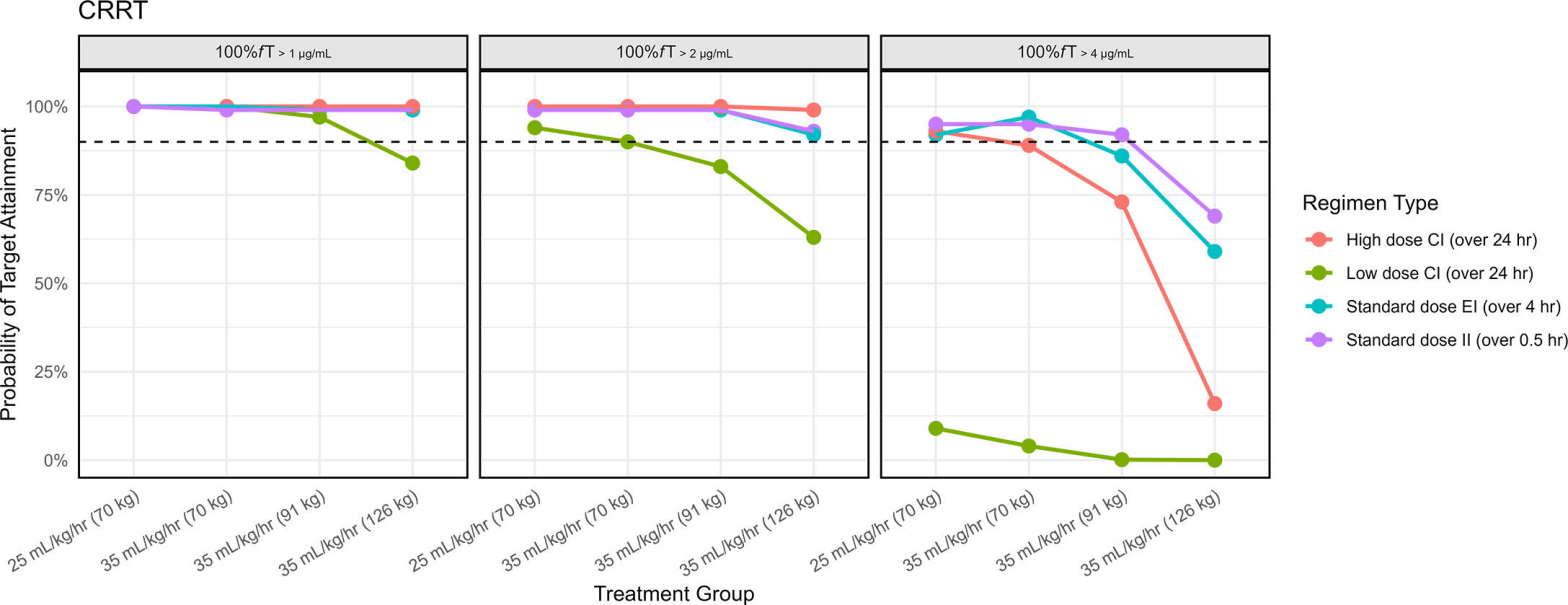
Probability of simulated unbound TAZ concentrations exceeding threshold values for II, EI, and CI by CRRT flow. Probability of simulated unbound TAZ concentrations achieving 100% *f*T_ > threshold_ for threshold targets of 1, 2, and 4 mg/L for II, EI, and CI regimens across renal function strata from 24 to 48 h after treatment is started. A dashed horizontal line indicates 90% target attainment. CI regimens are grouped as low (375–500 mg/day) or high (500–1,125 mg/day) dose depending on weight-based CRRT effluent rate. EI regimens (375–500 mg IV every 8–12 h over 4 h) and II regimens (375–500 mg IV every 6 h over 0.5 h) were designed to approximate HAP dosing across renal states.

Among patients on CRRT, all dosing regimens achieved ≥90% target attainment for 100% *f*T_>threshold_ for a threshold of 1 mg/L (Fig. 4) on day 2 of treatment, except for the low dose CI in patients weighing 126 kg at an effluent rate of 35 mL/kg/h. At a target of 2 mg/L, the low-dose CI regimen was inadequate for patients weighing ≥70 kg at an effluent rate of 35 mL/kg/h. At a target of 4 mg/L, the high-dose CI regimen was adequate only in patients weighing 70 kg at an effluent rate of 25 mL/kg/h. EI and II regimens performed well across targets of 1 and 2 mg/L. Failure to achieve a target of 4 mg/L was observed with EI regimens (≥91 kg) and II regimens (126 kg) at effluent rates of 35 mL/kg/h, suggesting a role for therapeutic drug monitoring (TDM) in larger patients on higher effluent flow rates.

## DISCUSSION

The final population PK model of TAZ suggested that CL was influenced by renal and non-renal clearance pathways among critically ill patients not requiring CRRT and by CRRT effluent flow rates among patients requiring CRRT. Our population PK model estimates for renal clearance are generally similar to prior studies in ICU patients with various degrees of renal dysfunction. Kalaria et al. found that total TAZ CL per 120 mL/min CrCL was 5.27 L/h after non-linear scaling (26), which is similar to our population estimate of 4.9 L/h and falls within the bootstrap 95% CI for our model (Table 1). Of note, after scaling CrCL to their sample mean of 78 mL/min, the average total TAZ CL would be 4.6 L/h. Solving for total TAZ clearance in our cohort for the average CrCL yielded 4.38 L/h. Reeder et al. estimated total TAZ CL among critically ill patients to be roughly 4 L/h in a population of patients with various degrees of renal impairment including those requiring CRRT, which is similar to our findings (27). However, Felton et al. estimated total TAZ CL to be roughly threefold greater among hospitalized adult patients with preserved renal function.(28) Among patients requiring CRRT, our model estimated total clearance to be 4 L/h standardized to an effluent flow rate of 2 L/h (i.e., 50% extracorporeal clearance), which is similar to prior estimates (11, 27). Because the determination of TAZ population PK parameters is sample dependent, simulations generated by a given population PK model should only be applied to demographically similar patients.

Kalaria et al. also evaluated TAZ PK/PD attainment and focused primarily on a single target of maintaining free TAZ concentrations above 2 mg/L for 85% of the dosing interval (26). This target is based on *in vitro* studies demonstrating a 2-log decrease in bacterial counts vs *E. coli* producing high levels of CTX-M extended-spectrum beta-lactamase. We studied a range of thresholds due to uncertainty concerning optimal TAZ PK/PD targets (9, 18, 29). We assessed 100% *f*T_>threshold_ in our models to reflect a conservative approach to empiric pneumonia treatment where infection is often caused by resistant Gram-negative pathogens, and because lab-derived thresholds may not always translate to patients.

Dosing regimens we evaluated differed from those evaluated by Kalaria et al. who simulated traditional 30-min infusions given every 6, 8, or 12 h (26), along with extended infusions lasting 4 or 6 h and continuous infusions up to 2 g/day. Their simulations did not include dose adjustments for patients receiving CRRT or those with very high renal clearance. In contrast, our study evaluated dosing regimens across a broad range of renal states, including CrCL up to 150 mL/min and CRRT. We tested intermittent infusions, extended infusions, and both low- and high-dose continuous infusions up to 1,500 mg/day, reflecting dosing strategies often used in critically ill patients with HAP and VAP. In our simulations, low-dose CI regimens often failed to reach at least 90% target attainment when higher thresholds (4 mg/L) were applied, especially in patients with supra-normal renal clearance at higher body weight individuals (>70 kg) at effluent flow rates of 35 mL/kg/h. High-dose CI and extended infusions performed better against a target of 2 mg/L, yet sometimes fell short in patients with very high CrCL. Patients requiring CRRT at higher effluent rates and those with preserved renal function may require TDM to optimize TAZ.

Optimizing treatment for infections caused by β-lactamase-producing Enterobacterales requires adequate TAZ exposure, particularly in severe infections such as HAP. Prior research suggests that traditional PK/PD indices may underestimate the complexity of β-lactam/β-lactamase inhibitor interactions. The MICi framework proposed by Abodakpi et al. addresses this by modeling the dynamic reduction of the partner antibiotic’s MIC as a function of inhibitor concentration, offering a more mechanistically relevant approach (30). However, this approach requires validation. The increased risk of clinical failure observed by Gatti et al. with suboptimal joint PK/PD attainment supports a need to assess both PIP and TAZ PK/PD (18). Our study suggests that attainment rates for more aggressive targets (e.g., 4 mg/L for an ESBL-producing isolate) may not be possible without a corresponding increase in PIP exposure above a threshold of 96 mg/L using a population approach (i.e., in the absence of TDM), based on our joint modeling analysis. TDM of TAZ may also prove helpful in identifying patients for whom TAZ is low in spite of adequate PIP levels.

Our study has several important limitations that should be considered when interpreting the results. First, the sample size was relatively small, with 35 patients contributing 162 plasma samples. We were not able to estimate parameters for all patient subgroups (e.g., limited sampling around dialysis requiring a fixed HD clearance), increasing the uncertainty in some model estimates. Therefore, we conducted rigorous bootstrap resampling to quantify uncertainty in all model parameters. However, the opportunistic nature of PK sampling in this study was adequate to characterize TAZ during the infusion period and early elimination phase as shown in the time after dose visual predictive check (Fig. 2). Forthcoming research will evaluate our model by external validation. Second, all patients included were critically ill ICU patients with HAP or VAP, so our findings may not generalize to patients with less severe illnesses or with infections at other sites. Third, while our analysis focused on how likely different dosing regimens were to achieve PK/PD targets, we were unable to link these simulations to clinical outcomes, such as microbiological cure, symptom improvement, or survival. Thus, the real-world benefits of meeting specific TAZ concentration thresholds remain uncertain, especially in antimicrobial-resistant isolates, such as ESBL-producing Enterobacterales. There is uncertainty regarding whether attaining tazobactam targets alone provides additional clinical benefit beyond achieving adequate piperacillin exposure. Fourth, our modeling approach relied on several assumptions that are common in population PK studies. For example, we used a one-compartment model and assumed that 70% of TAZ remains unbound in the blood. However, protein binding can vary widely in critically ill patients due to factors like inflammation and low albumin levels. Finally, the PD targets we used were derived from *in vitro* and animal studies involving various β-lactamases (e.g., TEM and CTX-M enzymes) expressed primarily in *E. coli*. These targets may not fully reflect optimal inhibitory activity against the full range of β-lactamase enzymes seen in clinical practice.

### Conclusion

We developed a population PK model of TAZ in critically ill patients with HAP, highlighting the impact of renal function and CRRT on drug clearance. Simulations showed standard dosing regimens achieved targets of 1–2 mg/L but often failed to maintain 4 mg/L, particularly in patients with high CrCL and those receiving high effluent flow rates (e.g., higher body weights). These findings support dose individualization based on renal function and suggest that TDM may be necessary in patients with augmented clearance and those individuals requiring CRRT.

## SUPPLEMENTAL MATERIAL

Data S1 aac.01766-25-s0001.xlsx List of SCRIPT Study Investigators acknowledged.

Supplemental material aac.01766-25-s0002.pdf Tables S1 and S2; Fig. S1 and S2.

ASM does not own the copyrights to Supplemental Material that may be linked to, or accessed through, an article. The authors have granted ASM a non-exclusive, world-wide license to publish the Supplemental Material files. Please contact the corresponding author directly for reuse.

## References

[1] Melsen WG, Rovers MM, Koeman M, Bonten MJM. 2011. Estimating the attributable mortality of ventilator-associated pneumonia from randomized prevention studies. Crit Care Med 39:2736–2742. doi:10.1097/CCM.0b013e3182281f33 21765351

[2] Ibn Saied W, Mourvillier B, Cohen Y, Ruckly S, Reignier J, Marcotte G, Siami S, Bouadma L, Darmon M, de Montmollin E, Argaud L, Kallel H, Garrouste-Orgeas M, Soufir L, Schwebel C, Souweine B, Glodgran-Toledano D, Papazian L, Timsit J-F. 2019. A comparison of the mortality risk associated with ventilator-acquired bacterial pneumonia and nonventilator ICU-acquired bacterial pneumonia*. Crit Care Med 47:345–352. doi:10.1097/CCM.0000000000003553 30407949

[3] Kollef MH, Nováček M, Kivistik Ü, Réa-Neto Á, Shime N, Martin-Loeches I, Timsit J-F, Wunderink RG, Bruno CJ, Huntington JA, Lin G, Yu B, Butterton JR, Rhee EG. 2019. Ceftolozane–tazobactam versus meropenem for treatment of nosocomial pneumonia (ASPECT-NP): a randomised, controlled, double-blind, phase 3, non-inferiority trial. Lancet Infect Dis 19:1299–1311. doi:10.1016/S1473-3099(19)30403-7 31563344

[4] Torres A, Zhong N, Pachl J, Timsit JF, Kollef M, Chen Z, Song J, Taylor D, Laud PJ, Stone GG, Chow JW. 2018. Ceftazidime-avibactam versus meropenem in nosocomial pneumonia, including ventilator-associated pneumonia (REPROVE): a randomised, double-blind, phase 3 non-inferiority trial. Lancet Infect Dis 18:285–295. doi:10.1016/S1473-3099(17)30747-8 29254862

[5] Martin-Loeches I, Timsit J-F, Kollef MH, Wunderink RG, Shime N, Nováček M, Kivistik Ü, Réa-Neto Á, Bruno CJ, Huntington JA, Lin G, Jensen EH, Motyl M, Yu B, Gates D, Butterton JR, Rhee EG. 2022. Clinical and microbiological outcomes, by causative pathogen, in the ASPECT-NP randomized, controlled, Phase 3 trial comparing ceftolozane/tazobactam and meropenem for treatment of hospital-acquired/ventilator-associated bacterial pneumonia. J Antimicrob Chemother 77:1166–1177. doi:10.1093/jac/dkab494 35022730 PMC9432134

[6] Henderson A, Paterson DL, Chatfield MD, Tambyah PA, Lye DC, De PP, Lin RTP, Chew KL, Yin M, Lee TH, et al.. 2021. Association between minimum inhibitory concentration, beta-lactamase genes and mortality for patients treated with piperacillin/tazobactam or meropenem from the MERINO study. Clin Infect Dis 73:e3842–e3850. doi:10.1093/cid/ciaa1479 33106863

[7] Harris PNA, Tambyah PA, Lye DC, Mo Y, Lee TH, Yilmaz M, Alenazi TH, Arabi Y, Falcone M, Bassetti M, et al.. 2018. Investigators MT, the Australasian society for infectious disease clinical research N. JAMA 320:984–994. doi:10.1001/jama.2018.12163 30208454 PMC6143100

[8] Tamma PD, Han JH, Rock C, Harris AD, Lautenbach E, Hsu AJ, Avdic E, Cosgrove SE, Antibacterial Resistance Leadership G. 2015. Carbapenem therapy is associated with improved survival compared with piperacillin-tazobactam for patients with extended-spectrum β-lactamase bacteremia. Clin Infect Dis 60:1319–1325. doi:10.1093/cid/civ003 25586681 PMC4462658

[9] Nicasio AM, VanScoy BD, Mendes RE, Castanheira M, Bulik CC, Okusanya OO, Bhavnani SM, Forrest A, Jones RN, Friedrich LV, Steenbergen JN, Ambrose PG. 2016. Pharmacokinetics-pharmacodynamics of tazobactam in combination with piperacillin in an in vitro infection model. Antimicrob Agents Chemother 60:2075–2080. doi:10.1128/AAC.02747-15 26787689 PMC4808219

[10] Strayer AH, Gilbert DH, Pivarnik P, Medeiros AA, Zinner SH, Dudley MN. 1994. Pharmacodynamics of piperacillin alone and in combination with tazobactam against piperacillin-resistant and -susceptible organisms in an in vitro model of infection. Antimicrob Agents Chemother 38:2351–2356. doi:10.1128/AAC.38.10.2351 7840569 PMC284743

[11] Valtonen M, Tiula E, Takkunen O, Backman JT, Neuvonen PJ. 2001. Elimination of the piperacillin/tazobactam combination during continuous venovenous haemofiltration and haemodiafiltration in patients with acute renal failure. J Antimicrob Chemother 48:881–885. doi:10.1093/jac/48.6.881 11733473

[12] Wyeth Pharmaceuticals Inc. 2017. Zosyn (piperacillin-tazobactam) [package insert]. Wyeth Pharmaceuticals Inc

[13] Dulhunty JM, Brett SJ, De Waele JJ, Rajbhandari D, Billot L, Cotta MO, Davis JS, Finfer S, Hammond NE, Knowles S, Liu X, McGuinness S, Mysore J, Paterson DL, Peake S, Rhodes A, Roberts JA, Roger C, Shirwadkar C, Starr T, Taylor C, Myburgh JA, Lipman J, BLING III Study Investigators. 2024. Continuous vs intermittent β-Lactam antibiotic infusions in critically ill patients with sepsis: the BLING iii randomized clinical trial. JAMA 332:629–637. doi:10.1001/jama.2024.9779 38864155 PMC11170452

[14] Vardakas KZ, Voulgaris GL, Maliaros A, Samonis G, Falagas ME. 2018. Prolonged versus short-term intravenous infusion of antipseudomonal β-lactams for patients with sepsis: a systematic review and meta-analysis of randomised trials. Lancet Infect Dis 18:108–120. doi:10.1016/S1473-3099(17)30615-1 29102324

[15] Rhodes NJ, Liu J, O’Donnell JN, Dulhunty JM, Abdul-Aziz MH, Berko PY, Nadler B, Lipman J, Roberts JA. 2018. Prolonged infusion piperacillin-tazobactam decreases mortality and improves outcomes in severely ill patients: results of a systematic review and meta-analysis. Crit Care Med 46:236–243. doi:10.1097/CCM.0000000000002836 29116995

[16] Abdul-Aziz MH, Hammond NE, Brett SJ, Cotta MO, De Waele JJ, Devaux A, Di Tanna GL, Dulhunty JM, Elkady H, Eriksson L, Hasan MS, Khan AB, Lipman J, Liu X, Monti G, Myburgh J, Novy E, Omar S, Rajbhandari D, Roger C, Sjövall F, Zaghi I, Zangrillo A, Delaney A, Roberts JA. 2024. Prolonged vs intermittent infusions of β-Lactam antibiotics in adults with sepsis or septic shock: a systematic review and meta-analysis. JAMA 332:638–648. doi:10.1001/jama.2024.9803 38864162 PMC11170459

[17] Cojutti PG, Pai MP, Tonetti T, Siniscalchi A, Viale P, Pea F. 2024. Balancing the scales: achieving the optimal beta-lactam to beta-lactamase inhibitor ratio with continuous infusion piperacillin/tazobactam against extended spectrum beta-lactamase producing Enterobacterales Antimicrob Agents Chemother 68:e0140423. doi:10.1128/aac.01404-23 38411995 PMC10994818

[18] Gatti M, Rinaldi M, Tonetti T, Siniscalchi A, Viale P, Pea F. 2023. Could an optimized joint pharmacokinetic/pharmacodynamic target attainment of continuous infusion piperacillin-tazobactam be a valuable innovative approach for maximizing the effectiveness of monotherapy even in the treatment of critically ill patients with documented extended-spectrum beta-lactamase-producing Enterobacterales bloodstream infections and/or ventilator-associated pneumonia? Antibiotics (Basel) 12:1736. doi:10.3390/antibiotics12121736 38136770 PMC10740629

[19] Zurawska M, Valadez A, Harlan E, Williamson R, Scheetz MH, Neely MN, Yarnold PR, Kang M, Donnelly HK, Martinez F, Korth E, Medernach RL, Nozick SH, Hauser AR, Ozer EA, Diaz E, Misharin AV, Wunderink RG, Rhodes NJ. 2025. Pharmacokinetic-pharmacodynamic target attainment with continuous infusion piperacillin in patients admitted to the ICU with hospital-acquired pneumonia. Antimicrob Agents Chemother 70:e01760-25. 10.1128/aac.01760-25. 41460103 PMC12888853

[20] Bjergum MW, Barreto EF, Scheetz MH, Rule AD, Jannetto PJ. 2021. Stability and Validation of a High-Throughput LC-MS/MS Method for the Quantification of Cefepime, Meropenem, and Piperacillin and Tazobactam in Serum. J Appl Lab Med 6:1202–1212. doi:10.1093/jalm/jfab036 34086904 PMC8464404

[21] Department of Health and Human Services Food and Drug Administration Center for Drug Evaluation and Research (CDER) Center for Veterinary Medicine (CVM). 2018. Bioanalytical Method Validation Guidance for Industry

[22] Johnson CA, Halstenson CE, Kelloway JS, Shapiro BE, Zimmerman SW, Tonelli A, Faulkner R, Dutta A, Haynes J, Greene DS. 1992. Single-dose pharmacokinetics of piperacillin and tazobactam in patients with renal disease. Clin Pharmacol Ther 51:32–41. doi:10.1038/clpt.1992.5 1310077

[23] Bergstrand M, Hooker AC, Wallin JE, Karlsson MO. 2011. Prediction-corrected visual predictive checks for diagnosing nonlinear mixed-effects models. AAPS J 13:143–151. doi:10.1208/s12248-011-9255-z 21302010 PMC3085712

[24] Cockcroft DW, Gault MH. 1976. Prediction of creatinine clearance from serum creatinine. Nephron 16:31–41. doi:10.1159/000180580 1244564

[25] Boselli E, Breilh D, Rimmelé T, Guillaume C, Xuereb F, Saux M-C, Bouvet L, Chassard D, Allaouchiche B. 2008. Alveolar concentrations of piperacillin/tazobactam administered in continuous infusion to patients with ventilator-associated pneumonia. Crit Care Med 36:1500–1506. doi:10.1097/CCM.0b013e318170ba21 18434883

[26] Kalaria SN, Gopalakrishnan M, Heil EL. 2020. A population pharmacokinetics and pharmacodynamic approach to optimize tazobactam activity in critically ill patients. Antimicrob Agents Chemother 64:e02093-19. doi:10.1128/AAC.02093-19 31871076 PMC7038264

[27] Reeder JA, Creech CB, Nation RL, Gu K, Nalbant D, Wu N, Jimenez-Truque N, Fissell W, Rolsma SL, Fishbane N, Kirkpatrick CMJ, Patel PC, Watanabe A, Landersdorfer CB, Winokur P, An G. 2025. Utilizing an opportunistic clinical study and population-based pharmacokinetic models to identify rational empiric dosing regimens for piperacillin-tazobactam in critically ill patients. J Clin Pharmacol 65:452–465. doi:10.1002/jcph.6161 39628093 PMC11938006

[28] Felton TW, Hope WW, Lomaestro BM, Butterfield JM, Kwa AL, Drusano GL, Lodise TP. 2012. Population pharmacokinetics of extended-infusion piperacillin-tazobactam in hospitalized patients with nosocomial infections. Antimicrob Agents Chemother 56:4087–4094. doi:10.1128/AAC.00521-12 22585219 PMC3421565

[29] Rodriguez CA, Agudelo M, Zuluaga AF, Vesga O. 2017. In vivo pharmacodynamics of piperacillin/tazobactam: implications for antimicrobial efficacy and resistance suppression with innovator and generic products. Int J Antimicrob Agents 49:189–197. doi:10.1016/j.ijantimicag.2016.10.011 27988068

[30] Abodakpi H, Chang K-T, Gao S, Sánchez-Díaz AM, Cantón R, Tam VH. 2019. Optimal piperacillin-tazobactam dosing strategies against extended-spectrum-β-lactamase-producing Enterobacteriaceae. Antimicrob Agents Chemother 63:e01906-18. doi:10.1128/AAC.01906-18 30530606 PMC6355564

