# Supplementary material for: Critical threshold target attainment rates for tazobactam combined with piperacillin among patients admitted to the ICU with hospital-acquired pneumonia
Source: Antimicrob Agents Chemother. 2025 Dec 29;70(2):e01766-25. doi: 10.1128/aac.01766-25 (PMC12888870; doi:10.1128/aac.01766-25)
Supplement: Supplemental material — Tables S1 and S2; Fig. S1 and S2. [file aac.01766-25-s0002.pdf]

Supplemental Material:

Table S1. Piperacillin-tazobactam structural and covariate model development build

Run	Model	-2*LL (IS)	BICc (IS)	Forward Δ OFV	Forward Referent	Backward Δ OFV	Backward Referent	Selection	Observation model	Comments
**1**	**1 compartment base**	2436.76	2515.52	NA	NA				**y1**comb1<br>**y2**comb1	Base model 1 comp, regressor, high RSE on additive pip
**2**	**Run 1 with updated error models**	2436.01	2509.03	-0.75	1			Basis	**y1**prop<br>**y2**comb1	Base model 1 comp based on run 1. Prop model pip, comb 1 for taz
**3**	**2 compartment base**	2399.58	2495.57	-36.43	2	11.37	5	Reject	**y1**prop<br>**y2**comb1	Base 2 comp model based on run 2. Unable to estimate peripheral V.
**4**	**Run 2 with CLR and CLNR**	2412.05	2503.67	-23.96	2	-1.1	5	Accept	**y1**prop<br>**y2**comb1	Base model 1 comp based on run 2 adding non-renal CL. High RSE on IIV and CLNR pop est.
**5**	**Run 4 with no IIV on CLNR**	2410.95	2495.46	-25.06	2	NA	NA	Final	**y1**prop<br>**y2**comb1	Base model 1 comp based on run 2 adding non-renal CL no IIV. Condition number 67
**6**	**Run 5 with combined error**	2412.14	2502.39	1.19	5				**y1**comb1<br>**y2**comb1	Base model 1 comp based on run 4 testing comb1 error all. BICC inc.
**7**	**Run 5 with proportional error**	2429.58	2508.34	18.63	5				**y1**prop<br>**y2**prop	Base model 1 comp based on run 4 testing prop error all, inc BICC.
**8**	**Run 5 with WT on V regressor**	2416.22	2500.73	5.27	5				**y1**prop<br>**y2**comb1	Base model 1 comp based on run 3 scaling V to WT/70. Condition number 43.9 BICC increased.
**9**	**Run 5 with allometric scaling**	2415.18	2499.69	4.23	5				**y1**prop<br>**y2**comb1	Base model 1 comp based on run 4 allometric WT/70. inc BICC and high RSE on all parameters.
**10**	**Run 5 with CRRT on Vd piecewise**	2411.76	2514.86	0.81	5				**y1**prop<br>**y2**comb1	Base model 1 comp based on run 4 adding piecewise V for CRRT. High RSE on Vs inc BICC.
**11**	**Run 5 with CRRT on Vd fractional change**	2411.3	2507.29	0.35	5				**y1**prop<br>**y2**comb1	Base model 1 comp based on run 4 adding linearlized V on CRRT. High RSE on V inc BICC

Table legend: Sequence of structural and error model development runs, including objective function values, model selection criteria, and key observations. For each run, the model type, changes implemented, and evaluation statistics are listed. Forward and backward inclusion steps are indicated along with the corresponding change in objective function value (ΔOFV) and referent model. The final selected model is noted.

Abbreviations: ΔOFV, change in objective function value; BICc (IS), corrected Bayesian Information Criterion by Importance Sampling; CLR, renal clearance; CLNR, non-renal clearance; IIV, inter-individual variability; Vd, volume of distribution; WT, body weight; CRRT, continuous renal replacement therapy; comb1, combined error model (additive + proportional error); prop; proportional error model; RSE, relative standard error; NA, not applicable.

Table S2. Dosing regimens and renal dispositions evaluated in simulations*

Tazobactam Regimens: Renal Disposition, Doses, and Intervals

Regimen	Renal Disposition	Dose (mg)	Interval
EI_CRRT_25_70kg	CRRT 25 mL/kg/hr	500	q12h
EI_CRRT_35_70kg	CRRT 35 mL/kg/hr	500	q8h
EI_CRCL_25_mL_min	CrCl 25 mL/min	375	q12h
EI_CRCL_50_mL_min	CrCl 50 mL/min	500	q8h
EI_CRCL_75_mL_min	CrCl 75 mL/min	500	q8h
EI_CRCL_150_mL_min	CrCl 150 mL/min	500	q6h
low_CI_CRRT_25_70kg	CRRT 25 mL/kg/hr	375	24h CI
low_CI_CRRT_35_70kg	CRRT 35 mL/kg/hr	500	24h CI
low_CI_CRCL_25_mL_min	CrCl 25 mL/min	375	24h CI
low_CI_CRCL_50_mL_min	CrCl 50 mL/min	500	24h CI
low_CI_CRCL_75_mL_min	CrCl 75 mL/min	750	24h CI
low_CI_CRCL_150_mL_min	CrCl 150 mL/min	1125	24h CI
high_CI_CRRT_25_70kg	CRRT 25 mL/kg/hr	750	24h CI
high_CI_CRRT_35_70kg	CRRT 35 mL/kg/hr	1000	24h CI
high_CI_CRCL_25_mL_min	CrCl 25 mL/min	500	24h CI
high_CI_CRCL_50_mL_min	CrCl 50 mL/min	1000	24h CI

Tazobactam Regimens: Renal Disposition, Doses, and Intervals

Regimen	Renal Disposition	Dose (mg)	Interval
high_CI_CRCL_75_mL_min	CrCl 75 mL/min	1125	24h CI
high_CI_CRCL_150_mL_min	CrCl 150 mL/min	1500	24h CI
II_CRRT_25_70kg	CRRT 25 mL/kg/hr	375	q8h
II_CRRT_35_70kg	CRRT 35 mL/kg/hr	375	q6h
II_CRCL_25_mL_min	CrCl 25 mL/min	375	q6h
II_CRCL_50_mL_min	CrCl 50 mL/min	500	q6h
II_CRCL_75_mL_min	CrCl 75 mL/min	500	q6h
II_CRCL_150_mL_min	CrCl 150 mL/min	500	q6h
II_CRRT_35_91kg	CRRT 35 mL/kg/hr	500	q6h
EI_CRRT_35_91kg	CRRT 35 mL/kg/hr	500	q8h
CI_CRRT_35_91kg	CRRT 35 mL/kg/hr	1125	24h CI
II_CRRT_35_126kg	CRRT 35 mL/kg/hr	500	q6h
EI_CRRT_35_126kg	CRRT 35 mL/kg/hr	500	q8h
CI_CRRT_35_126kg	CRRT 35 mL/kg/hr	1125	24h CI
low_CI_CRRT_35_91kg	CRRT 35 mL/kg/hr	500	24h CI
low_CI_CRRT_35_126kg	CRRT 35 mL/kg/hr	500	24h CI

***Footnote:** Covariates / regressors were fixed at each defined category while the population parameters and their uncertainties were sampled using the full covariance

matrix of the population parameters (fixed effects) and their corresponding inter-individual variability estimates (random effects).

Figure S1. Individual weighted residuals of tazobactam in plasma

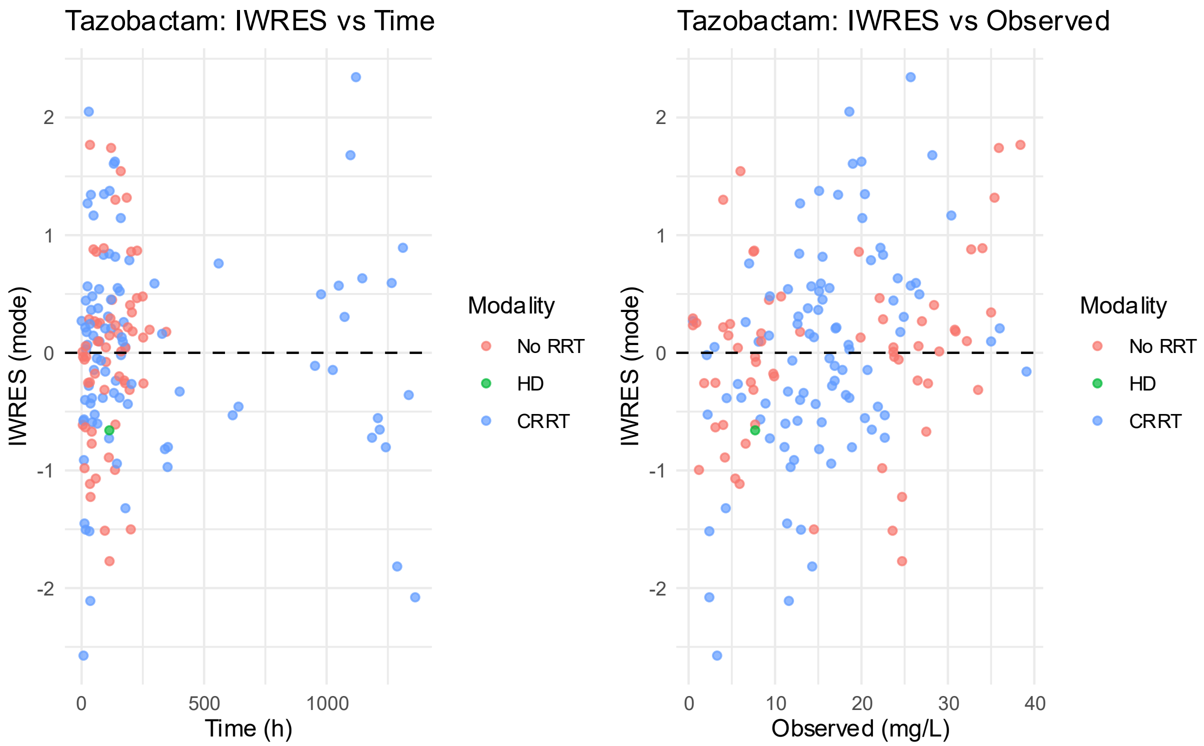

Figure legend: Observed time of sample collection vs. residual (left) and observed plasma concentrations of tazobactam (DV) vs. residual (right). Modal Empirical Bayes Estimates were used to generate individual weighted residuals (IWRES).

Figure S2. Representative simulated tazobactam plasma concentrations over the first 48 hours with high and low dose CI dosing regimens.

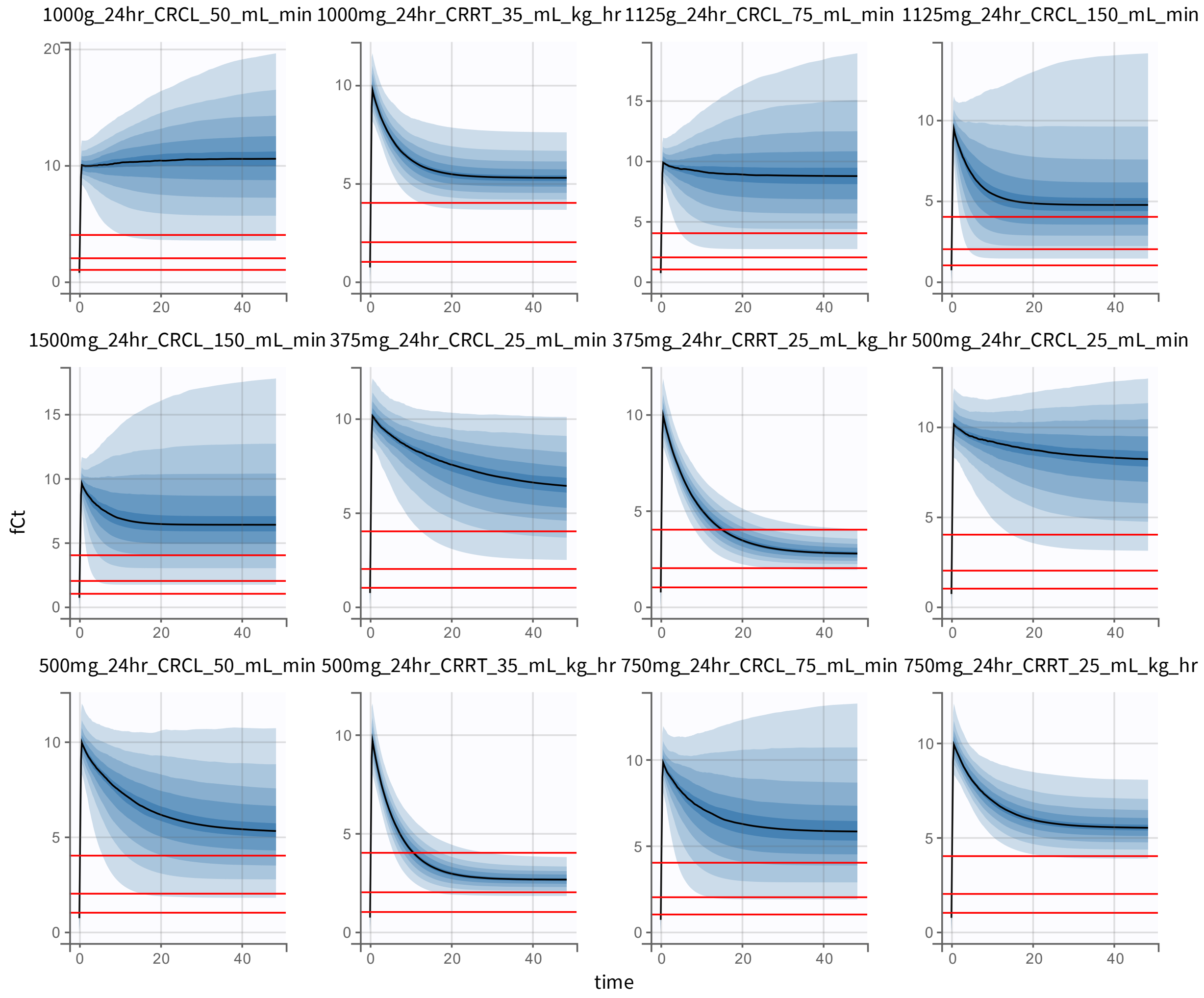

Figure legend: Simulated tazobactam (TAZ) concentration distributions were generated. TAZ concentrations from 24 to 48 hr were assessed based on attainment of 100% $fT_{> \text{threshold}}$ for threshold targets of 1, 2, and 4 mg/L. CI regimens are grouped as low (375–1125 mg/day IV over 24 hr) or high (750–1500 mg/day). Figure includes visual cues (red lines) added to distinguish TAZ concentrations of 1, 2, and 4 mg/L.
